# A Blinded Evaluation of Brain Morphometry for Differential Diagnosis of Atypical Parkinsonism

**DOI:** 10.1002/mdc3.13987

**Published:** 2024-02-05

**Authors:** Kazuya Kawabata, Florian Krismer, Beatrice Heim, Anna Hussl, Christoph Mueller, Christoph Scherfler, Elke R. Gizewski, Klaus Seppi, Werner Poewe

**Affiliations:** ^1^ Department of Neurology Medical University Innsbruck Innsbruck Austria; ^2^ Department of Neurology Nagoya University Graduate School of Medicine Nagoya Japan; ^3^ Neuroimaging Research Core Facility Medical University Innsbruck Innsbruck Austria; ^4^ Department of Neuroradiology Medical University Innsbruck Innsbruck Austria

**Keywords:** atypical parkinsonism, differential diagnosis, multiple system atrophy, progressive supranuclear palsy, voxel based morphometry

## Abstract

**Background:**

Advanced imaging techniques have been studied for differential diagnosis between PD, MSA, and PSP.

**Objectives:**

This study aims to validate the utility of individual voxel‐based morphometry techniques for atypical parkinsonism in a blinded fashion.

**Methods:**

Forty‐eight healthy controls (HC) T1‐WI were used to develop a referential dataset and fit a general linear model after segmentation into gray matter (GM) and white matter (WM) compartments. Segmented GM and WM with PD (n = 96), MSA (n = 18), and PSP (n = 20) were transformed into *z*‐scores using the statistics of referential HC and individual voxel‐based *z*‐score maps were generated. An imaging diagnosis was assigned by two independent raters (trained and untrained) blinded to clinical information and final diagnosis. Furthermore, we developed an observer‐independent index for ROI‐based automated differentiation.

**Results:**

The diagnostic performance using voxel‐based z‐score maps by rater 1 and rater 2 for MSA yielded sensitivities: 0.89, 0.94 (95% CI: 0.74–1.00, 0.84–1.00), specificities: 0.94, 0.80 (0.90–0.98, 0.73–0.87); for PSP, sensitivities: 0.85, 0.90 (0.69–1.00, 0.77–1.00), specificities: 0.98, 0.94 (0.96–1.00, 0.90–0.98). Interrater agreement was good for MSA (Cohen's kappa: 0.61), and excellent for PSP (0.84). Receiver operating characteristic analysis using the ROI‐based new index showed an area under the curve (AUC): 0.89 (0.77–1.00) for MSA, and 0.99 (0.98–1.00) for PSP.

**Conclusions:**

These evaluations provide support for the utility of this imaging technique in the differential diagnosis of atypical parkinsonism demonstrating a remarkably high differentiation accuracy for PSP, suggesting potential use in clinical settings in the future.

The differential diagnosis of atypical parkinsonian syndromes, including multiple system atrophy (MSA) and progressive supranuclear palsy (PSP), from Parkinson's disease (PD) is challenging during the early stages of the disease, since these conditions may present with overlapping clinical features in the early phase.^1^, ^2^ Specifically, MSA may feature varying degrees of parkinsonism, cerebellar ataxia, and autonomic failure, and can be classified into two motor phenotypes: parkinsonian variant (MSA‐P), characterized by striatonigral degeneration, and cerebellar variant (MSA‐C), associated with olivopontocerebellar cell loss.^2^, ^3^ Richardson's type PSP is typically characterized by supranuclear vertical gaze palsy, postural instability with falls, bradykinesia, and axial rigidity; however, PSP may present with several other phenotypes as has been acknowledged in the recent revision of PSP diagnostic criteria and may include a pure akinesia and gait freezing, a PD‐like, a corticobasal syndrome, and a frontal‐executive phenotype.^4^, ^5^ Nevertheless, an accurate diagnosis during the early stage is crucial for appropriate patient counseling and identifying homogenous patient cohorts for clinical trial.^6^

For differentiating parkinsonism, continuous clinical examination and monitoring, including the response to treatment, remain essential, but biomarker support using molecular or imaging markers play an important role. Conventional MRI studies have demonstrated classical findings in MSA and PSP, such as the “hot cross bun” or the “hummingbird” signs.^7^ However, these findings are not consistently present in all patients, limiting the utility of conventional MRI in the differential diagnostic process. Advanced MRI techniques, such as high resolution T1 weighted imaging (T1‐WI), diffusion‐weighted imaging (DWI), susceptibility‐weighted imaging (SWI), and machine learning methods have shown promise in improving diagnostic accuracy in the research field.^8^, ^9^, ^10^, ^11^, ^12^, ^13^, ^14^ Despite these advances, advanced methods are not yet widely applied in clinical settings and often lack validation in independent cohorts eliciting concerns on the generalizability of these advanced methods.

In this study, we applied the individual voxel‐based morphometry adjusting covariates (iVAC) system to the anatomical T1‐WI, an easy‐to‐process anatomical analysis pipeline introduced in 2021.^15^ This method enables visualization of individual statistical *z*‐score brain maps relative to healthy controls using color gradation, making it easier to recognize regional atrophic changes with potential for clinical application. The previous paper reported a high sensitivity in detecting anatomical abnormalities in the putamen, pons, middle cerebellar peduncle in MSA patients as well as high accuracy in differentiating MSA from PD. In the present study, we aimed to validate this method in an independent cohort as has been recommended in guidelines on the development of biomarkers. While the previous report only examined MSA and PD patients, in the present work, we also sought to investigate the diagnostic potential of this method in patients with PSP. We designed this study with two independent approaches: (1) a voxel‐based *z*‐score visualization map diagnosis by raters blinded to the clinical information and final diagnosis; and (2) a region‐of interest (ROI)‐based automated and observer‐independent index encompassing all relevant brain structure to facilitate the clinical applicability of the presented method.

## Methods

### Participants

This study includes MRI images of 134 patients clinically diagnosed with parkinsonism, including PD (n = 96), MSA (n = 18; MSA‐P: n = 12 and MSA‐C: n = 6), and PSP (n = 20; Richardson's syndrome: n = 11; PSP‐Parkinsonism: n = 9). All patients met clinical criteria for “probable” disease as defined in corresponding sets of diagnostic criteria,^4^, ^16^, ^17^ where MRI findings are not required, and were recruited as part of prospective biomarker studies between December 2011 and May 2013.^18^, ^19^ For the analysis pipeline, we also used 48 images from healthy controls as a referential database (age: mean ± standard deviation (SD) = 57.9 ± 11.8; female: n = 27; see the “Processing with Individual voxel‐based morphometry adjusting covariates (iVAC)” subsection below).

The study was approved by the Ethics Committee of the Medical University of Innsbruck. Participants’ written informed consent was obtained prior to study inclusion.

### MRI Protocol

Participants underwent 3.0‐T MR scanner (Magnetom Verio, Siemens, Erlangen, Germany) with a 12‐channel head coil. The parameters for the coronal T1‐weighted 3‐dimensional magnetization‐prepared rapid gradient echo (MPRAGE) were: TR = 1800 ms; TE = 2.18 ms; inversion time (TI) = 900 ms; slice thickness = 1.2 mm; matrix = 256 × 204 pixels; number of excitations = 1; flip angle = 9°; field of view, 220 × 165 mm.

### Preprocessing with CAT12

All images were preprocessed using the pipeline implemented in a Computational Anatomy Toolbox (CAT) for SPM12 (version CAT12.8.2, https://neuro-jena.github.io/cat/; SPM12, https://www.fl.ion.ucl.ac.uk/spm/software/spm12/) running on MATLAB (R2022b; MathWorks, Natick, MA, USA). In the CAT12 toolbox, T1‐WI was segmented into gray matter compartment (GM), white matter compartment (WM), and cerebrospinal fluid (CSF). The segmented images were normalized to the Montreal Neurological Institute (MNI) space using the Diffeomorphic Anatomical Registration Through Exponentiated Lie Algebra (DARTEL) algorithm. The normalized images were then resampled to an isotropic voxel resolution of 1.5 × 1.5 × 1.5 mm^3^. The resulting images were smoothed using an 8‐mm full‐width‐at‐half‐maximum 3‐dimensional Gaussian filter with SPM12, which was the same as the previous report.^15^ This segmentation takes approximately 15 min to 1 hr per subject, depending on settings.

### Processing with Individual Voxel‐Based Morphometry Adjusting Covariates (iVAC)

The iVAC is a toolbox for SPM12 (https://amrc.iwate-med.ac.jp/en/project-2/download/). Detailed explanations of the statistical process were described previously.^15^ In summary, the process was divided into following the steps.

1. Creation of a referential database from healthy controls (HC)

A referential dataset was generated only using HC participants’ images. In this analysis, a general linear model was applied, incorporating age, sex, and total intracranial volume (TIV) as explanatory variables. Brain maps, consisting of voxel‐level coefficients and residuals matrices, were then generated for the next step. Creating a referential database takes several to dozens of minutes to complete.

2 Transformation of segmented images into *z*‐score maps using HC statistics

Using the constructed referential database, individual voxel values of PD, MSA, and PSP patients for both GM and WM compartments were transformed into *z*‐scores, creating *z*‐score brain maps for GM and WM, taking into account age, sex, and TIV. The *z*‐scores were inverted to indicate that a higher z‐score represents the presence of atrophic changes.

3 Visualization of voxel‐based z‐score maps (for approach 1)

These resulting voxel‐based *z*‐score maps can be visualized with color gradation reports overlays on the standard brain. In this study, we set a *z*‐score threshold of 2 and only visualized voxels above this threshold, since a *z*‐score above 2 indicates that atrophic changes are observed in fewer than approximately 2.5% of the HCs. This approach allows us to focus on significantly atrophic regions.

4 Extraction of *z*‐scores within ROI (for approach 2)

The iVAC also outputs the mean voxel *z*‐score values within ROIs, which are described below.

In the analysis, any regions that were not part of the gray or white matter were excluded by masking them out. Transforming voxels into z‐scores and extracting ROI values takes a few to several minutes per step, depending on the number of images to transform, as conducted using a graphical user interface (GUI).

### Approach 1: Visual Inspection and Blind Diagnostic Decision with *z*‐Score Map Reports

The iVAC toolbox generates individual reports with color gradation *z*‐score maps overlaid onto template images. We thresholded GM and WM *z*‐score maps at a *z*‐score ≥2, indicating significant atrophic changes outside the 95% confidence intervals of healthy controls.

The two raters (K.K., B.H.) each assessed iVAC *z*‐score map reports for both GM and WM, generated from the anonymized T1‐WI (n = 134) of patients clinically diagnosed with MSA, PSP, and PD, and assigned a random ID. These were provided by the experienced neurologist (F.K.). The first rater (K.K.) had prior experience in using iVAC maps to differentiate MSA from PD from previously published work,^15^ while the second rater (B.H.) evaluated iVAC maps without any prior training. The raters conducted a blind diagnostic assessment without any clinical information based on individual reports, classifying patients as having MSA, PSP, or PD. Specifically, the raters focused on color gradations, particularly in the putamen and cerebellum for GM, and the pons, middle cerebellar peduncle (MCP), cerebellar white matter, and midbrain for WM reports. After the evaluation, the clinical diagnoses were opened and verified for consistency. The Cohen's kappa was then calculated for the agreement of two raters.

### Approach 2: Automated ROI‐Based Classification with Observer‐Independent Index

The Neuromorphometrics atlas (http://www.neuromorphometrics.com) and the Eve white matter atlas^20^ (https://github.com/Jfortin1/EveTemplate) were used for ROI‐level *z*‐score analysis.^15^ The Neuromorphometrics atlas is included in the CAT12 software. The Eve atlas, a WM parcellation map, was co‐registered and resliced using SPM12 to fit the preprocessed T1‐WI images. ROI‐level *z*‐scores were calculated as the mean of the voxel *z*‐scores within each ROI. For the ROI analysis, we used the GM *z*‐scores in the right and left putamen volumes from the Neuromorphometrics atlas, as well as WM *z*‐scores in the right and left midbrain and the right and left MCP from the Eve atlas. These ROI areas were selected based on relevant brain structures with MSA and PSP.

We generated a new index for differentiating MSA from others (PD and PSP) and PSP from others (PD and MSA) using Z_msa_ and Z_psp_ values. Z_psp_ represents the higher side of midbrain z‐score, the region involved in PSP, and Z_msa_ represents the highest *z*‐score value of the putamen and MCP, regions that may be involved in either MSA‐P or MSA‐C, or in both subtypes. As described above, the *z*‐score was inverted; therefore, higher Z_psp_ and Z_msa_ values are considered indicative of more atrophic changes. The Index was defined as follows:

$$Index\;=\;\left\{\begin{array}{llll} Z_{psp} & if\;Z_{psp}>0 & and & Z_{psp}\geq Z_{msa},\\ ‐Z_{msa} & if\;Z_{msa}>0 & and & Z_{psp}<Z_{msa},\\ 0 & if\;Z_{psp}\leq 0 & and & Z_{msa}\leq 0. \end{array}\right.\;$$

In summary, to define the Index for differentiation, we used the following flow:

1. *Z*_psp_ extracts higher midbrain *z*‐score, and *Z*_msa_ extracts the highest *z*‐score value of the putamen and MCP.
2. If both *Z*_psp_ and *Z*_msa_ are negative, the Index is set to 0.
3. If the *Z*_psp_ is greater than *Z*_msa_ (*Z*_psp_ ≥ *Z*_msa_), the Index corresponds to the midbrain z‐score (*Z*_psp_).
4. If the *Z*_psp_ is smaller than *Z*_msa_ (*Z*_psp_ < *Z*_msa_), the Index represents the negative of the highest *z*‐score of either putamen or MCP.

By using the Index, we can assess which region is more affected, either the midbrain or putamen/MCP, in the context of PSP and MSA.

### Statistical Analysis

Differences in demographics were assessed using the Kruskal‐Wallis test for age, disease duration, and Hoehn‐Yahr stage, and the chi‐square test for sex. Post‐hoc analysis was performed using the Mann–Whitney U test, with the *P* values corrected for multiple comparisons using Bonferroni correction.

For individual *z*‐scores of ROIs, we performed a pairwise Mann–Whitney U test to compare *z*‐score differences between two groups shown in Figure 4. The *P*‐values were corrected using Bonferroni correction for multiple comparisons for each ROI.

Receiver operating characteristic (ROC) analyses were conducted to differentiate MSA versus others, and PSP versus others with the Index described above. To estimate the optimal cutoff point, we applied the Youden Index (Youden's J), which is defined as J = sensitivity + specificity −1. DeLong's method was applied to calculate the 95% confidence interval (CI) for ROC curve.

### Validation for the Cutoffs from ROC Analyses

To validate the optimal cutoffs in the ROC analyses, we calculated leave‐one‐out cross‐validation (LOOCV) accuracy and Cohen's kappa. ROC and LOOCV analyses were performed using pROC and caret packages running on R version 4.3.1.

## Results

### Demographics

The demographics of participants are shown in Table S1. There were no significant differences in age or sex among the PD, MSA, and PSP groups. While disease duration was shorter and Hoehn & Yahr stage was higher in MSA and PSP compared with PD, no differences were observed between MSA and PSP.

### *Z*‐Score Map Appearance and Visual Decision of Diagnosis for MSA and PSP

**Figure 1:**
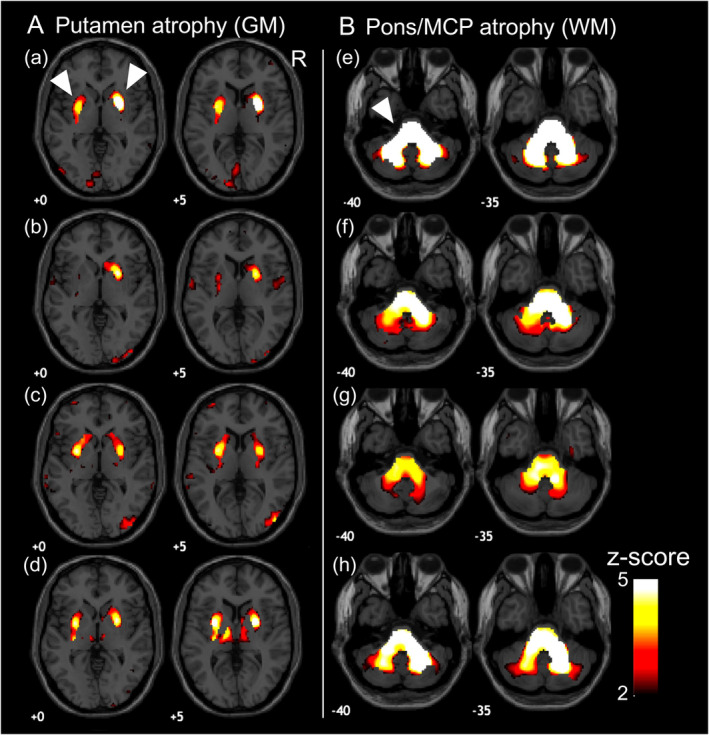
Typical Appearance of MSA in Putamen and Pons/MCP. This figure presents images with gray matter (A) and white matter (B) *z*‐score maps. (a)–(h) correspond to individual participants with MSA per letter. The red‐yellow color gradient represents atrophic regions with a *z*‐score >2, where atrophic changes occur in fewer than approximately 2.5% of the healthy controls. The number located in the bottom left of each normalized brain indicates the *Z* plane value in the Montreal Neurological Institute (MNI) coordinates. Panel (A) displays the typical appearance in MSA with the putamen atrophic changes (arrows). Panel (B) shows the typical appearance in MSA characterized by atrophy in the pons and middle cerebellar peduncle (MCP) (arrows). GM represents gray matter, and WM represents white matter. R represents the right side.

**Figure 2:**
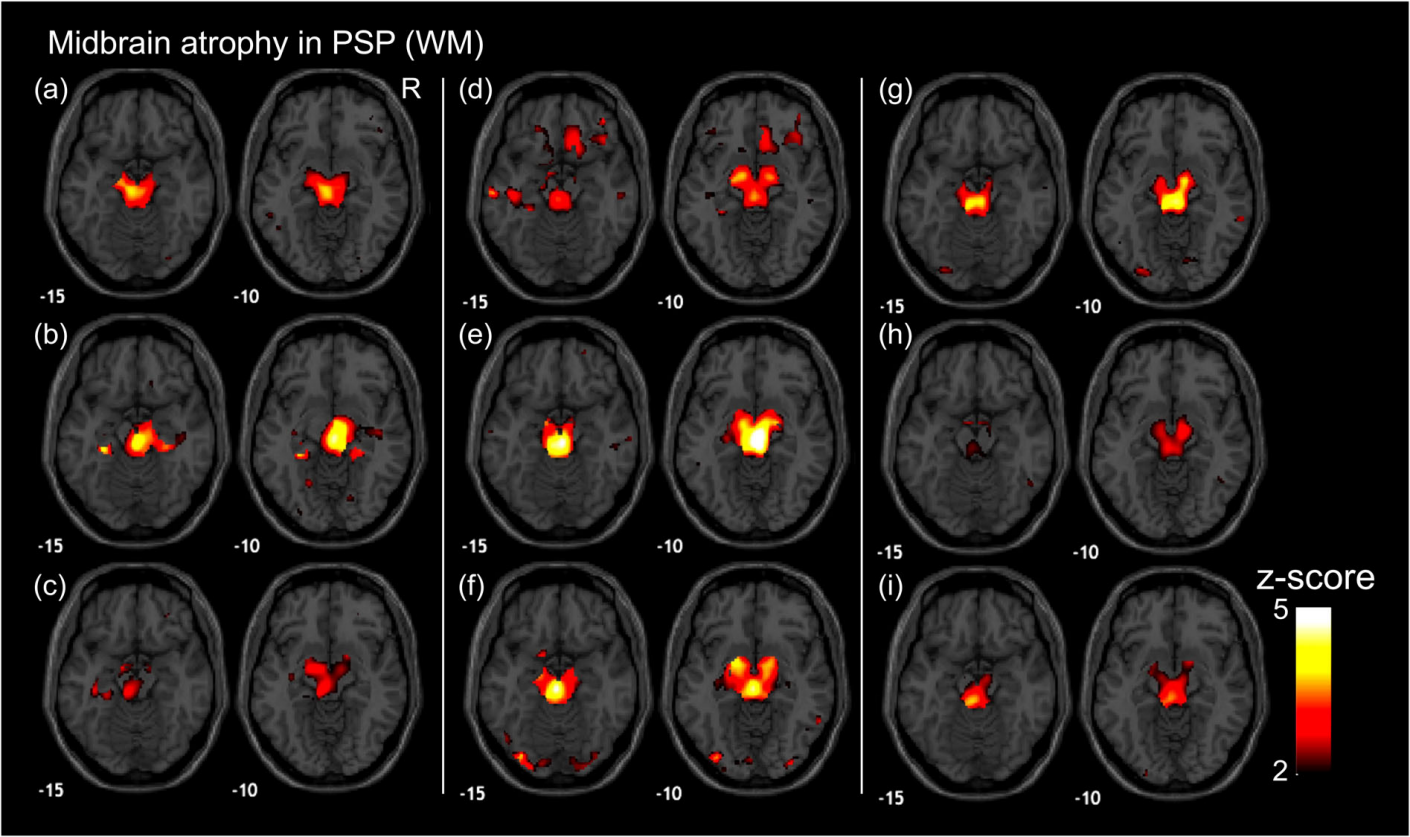
Typical Appearance of PSP in the Midbrain. This figure shows the images with white matter z‐score maps with typical appearance in PSP with the midbrain atrophic changes. (A)–(I) correspond to individual participants with PSP per letter. Red‐yellow color gradient shows atrophic regions with *z*‐score >2. The number located in the bottom left of each normalized brain indicates the *Z* plane value in the Montreal Neurological Institute (MNI) coordinates. WM represents white matter. R represents the right side.

Typical changes in MSA and PSP are illustrated in Fig. 1 and 2. In MSA, atrophy in the putamen presented especially for the MSA‐P subtype, while atrophy in the pons and MCP exhibited especially for MSA‐C. Blind diagnosis results of rater 1 (trained) showed that sensitivity is 0.889 (95%CI: 0.744–1.000), specificity 0.940 (0.896–0.975), and an accuracy 0.933 (0.890–0.975) for MSA, whereas ratings from rater 2 (untrained) yielded a sensitivity of 0.944, a specificity 0.802, and an overall accuracy 0.821 (0.756–0.886). The inter‐rater agreement was good with a Cohen's kappa of 0.614 (Table 1A).

**TABLE 1:** Contingency table of iVAC‐supported rater decisions and clinical diagnoses

	Rater 1			Rater 2		
A. MSA	Clinical Diagnosis			Clinical Diagnosis		
	MSA	Others	Total	MSA	Others	Total
iVAC‐based Diagnosis						
MSA	16	7	23	17	23	40
Others	2	109	111	1	93	94
Total	18	116	134	18	116	134
Sensitivity (95% CI)	0.889 (0.744–1.000)			0.944 (0.839–1.000)		
Specificity (95% CI)	0.940 (0.896–0.983)			0.802 (0.729–0.874)		
Accuracy (95% CI)	0.933 (0.890–0.975)			0.821 (0.756–0.886)		
Cohen's Kappa	0.614					

B. PSP	Clinical Diagnosis			Clinical Diagnosis		
	PSP	Others	Total	PSP	Others	Total
iVAC‐based Diagnosis						
PSP	17	2	19	18	7	25
Others	3	112	115	2	107	109
Total	20	114	134	20	114	134
Sensitivity (95% CI)	0.850 (0.694–1.000)			0.900 (0.769–1.000)		
Specificity (95% CI)	0.982 (0.958–1.000)			0.939 (0.895–0.983)		
Accuracy (95% CI)	0.963 (0.931–0.995)			0.933 (0.890–0.975)		
Cohen's Kappa	0.837					

*Note*: In Table 1A, “Others” includes both Parkinson's disease (PD) and Progressive Supranuclear Palsy (PSP). In Table 1B, “Others” refers to both PD and Multiple System Atrophy (MSA). iVAC, individual voxel‐based morphometry adjusting covariates.

**Figure 3:**
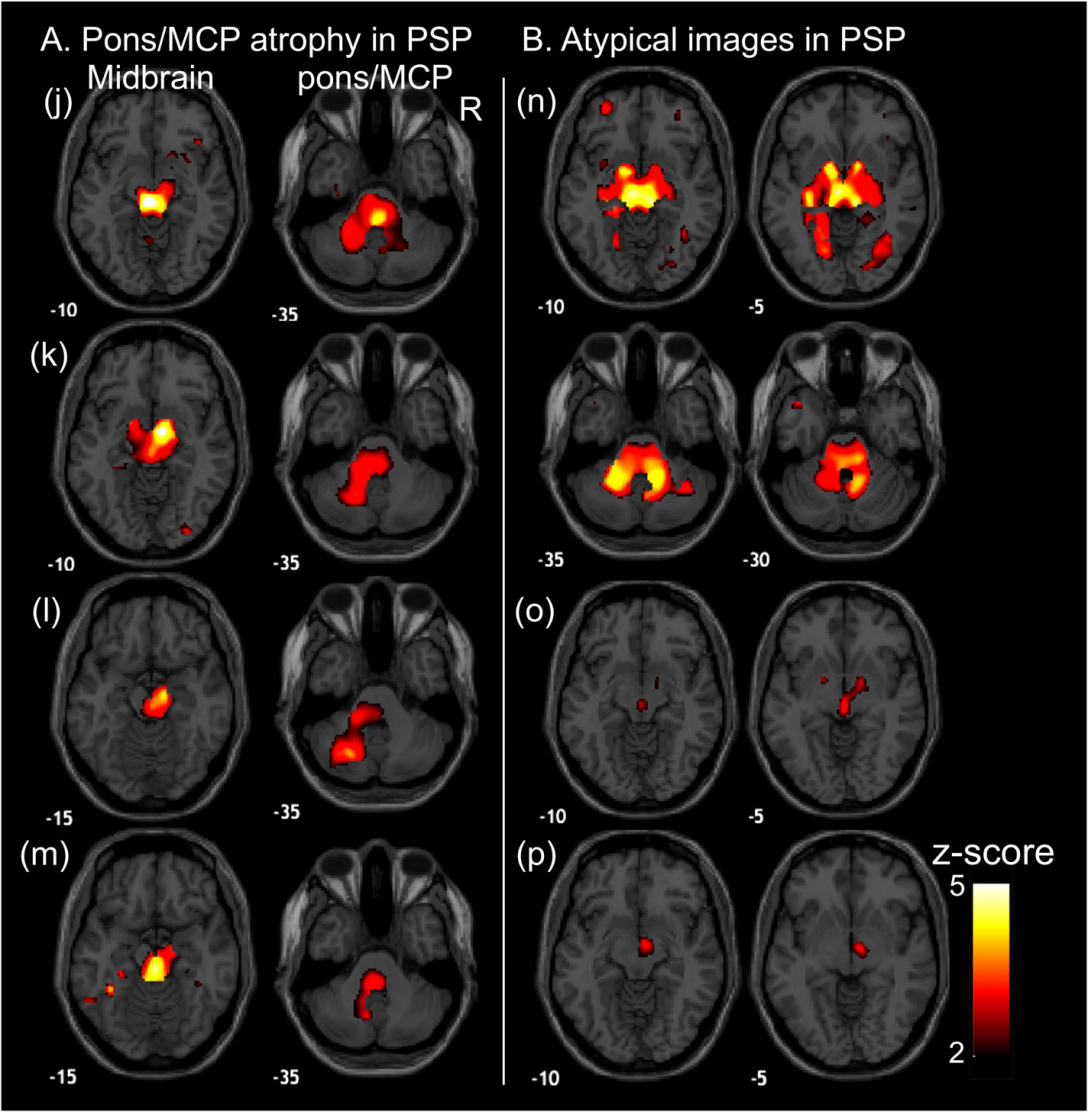
Other appearances in PSP Patients. This figure presents images featuring white matter *z*‐score maps with other appearance in PSP. The red‐yellow color gradient shows atrophic regions with *z*‐score >2. Panel (A) displays the pons and middle cerebellar peduncle (MCP) atrophy as well as the midbrain shown in the PSP patients. Panel (B) presents the patients with PSP misclassified into other diagnosis. (n) exhibited significant atrophic changes in both midbrain and pons/MCP atrophic changes with high *z*‐scores. Patients (o) and (p) showed smaller midbrain atrophic changes compared to other PSP patients, although atrophic changes in the midbrain were still present. R represents the right side.

In PSP, the most common change was a higher *z*‐score in the midbrain, indicating more pronounced midbrain atrophy (Fig. 2). This pattern was observed in 18 out of 20 patients with PSP. Moreover, five patients with PSP also displayed the pons/MCP atrophy, similar to the changes of the features in MSA‐C (Fig. 3A), although three out of five exhibited lateral changes (Fig. 3B, n). Additionally, two PSP patients also exhibited less obvious atrophic changes in the midbrain (Fig. 3B, o,p, and Table 1B). Overall, the sensitivity is 0.850 (95% CI: 0.694–1.000), specificity 0.982 (0.958–1.000), and accuracy 0.963 (0.931–0.995) for rater 1, and the sensitivity 0.900 (0.769–1.000), specificity 0.939 (0.895–0.983), and accuracy 0.933 (0.890–0.975) for rater 2. The inter‐rater agreement was excellent with a Cohen's kappa of 0.837.

### Automated ROI‐Based Classification Using the Putamen, MCP, and Midbrain

**Figure 4:**
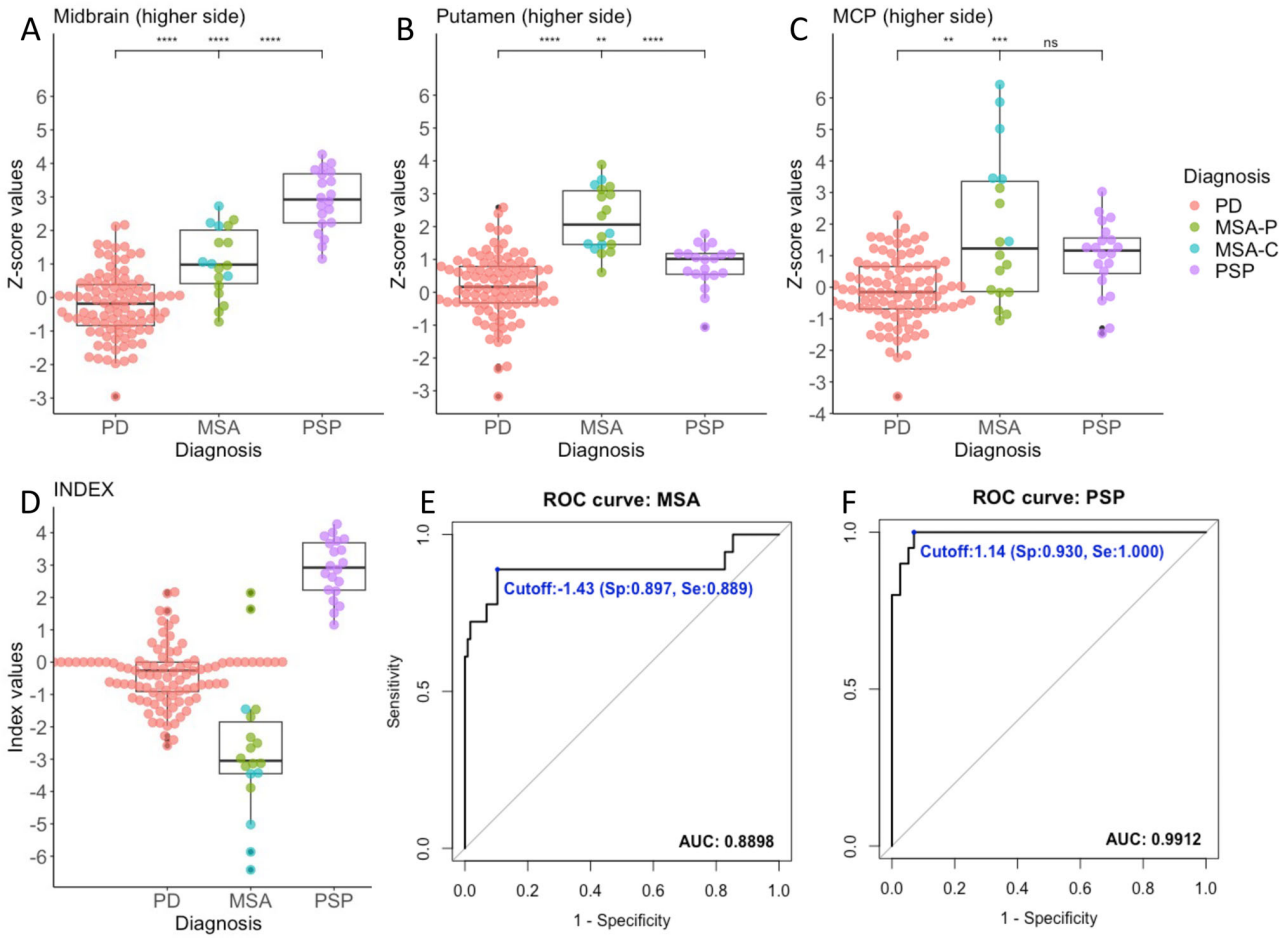
*Z*‐scores, Index values, and ROC analysis. Panels (A–C) show dotplots with boxplots of the highest *z*‐score values per participant for each ROI in the midbrain, putamen, and middle cerebellar peduncle (MCP). Higher *z*‐scores represent more atrophic changes. In the MSA group, bright green indicates MSA‐P, and medium turquoise indicates MSA‐C. **P < 0.01, ***P < 0.001, ****P < 0.0001, with *P* values calculated using Mann–Whitney U test, adjusted using Bonferroni correction for multiple comparisons. Panel (D) displays dotplots with boxplots of the index values we examined. The index represents the higher side of the midbrain and the highest *z*‐scores of the putamen and MCP, focusing only on positive *z*‐score values. Positive index values indicate that the *z*‐score of the midbrain is higher than that of the putamen and MCP, representing greater atrophic changes in the midbrain. Negative index values indicate that the *z*‐score of the putamen or MCP is higher than that of the midbrain, suggesting more atrophic changes in the putamen or MCP than in the midbrain. Panels (E) and (F) show the receiver operating characteristic (ROC) curves for differentiating MSA (Panel E) and PSP (Panel F). The cutoff indicates the optimal cutoff value identified using the Youden Index (J) method. Sp represents specificity, Se represents sensitivity, and AUC represents the area under the curve.

The *z*‐score of ROIs, where a higher z‐score represents greater atrophy (refer to the “Processing with Individual Voxel‐Based Morphometry Adjusting Covariates (iVAC)” subsection in Methods section), on the higher *z*‐score side of the midbrain, putamen, and MCP are shown in Figure 4A–C.

The plots of index we created are shown in Fig. 4D (see Methods for creating the index). The index represents the higher side of midbrain and the highest *z*‐scores of putamen and MCP. Positive index values suggest greater atrophic changes in the midbrain compared to the putamen/MCP. In contrast, negative index values indicate a higher *z*‐score in the putamen or MCP than in the midbrain, indicating more pronounced atrophic changes in the putamen/MCP than in the midbrain. From this perspective, all PSP participants displayed positive index values greater than 1. On the other hand, all but two in MSA patients showed negative values. The two MSA patients with positive indices had z‐scores in the putamen (higher side) of 1.18 and 0.61, MCP of −0.08 and −0.16, and midbrain of 2.14 and 1.64.

### ROC and Cross Validation Analyses

The ROC analysis for MSA showed that the optimal cutoff is −1.43 corresponding to the highest Youden's J, with a sensitivity of 0.889 (95% CI: 0.667–1.000), specificity of 0.897 (0.845–1.000), and AUC of 0.890 (0.765–1.000) (Fig. 4E). The LOOCV using this cutoff exhibited an accuracy of 0.896 (0.831–0.942) and a Cohen's Kappa of 0.636. In PSP patients, the optimal cutoff value was 1.14, resulting in a sensitivity of 1.000 (0.950–1.000) and a specificity of 0.930 (0.894–0.947). The AUC for the ROC curve was 0.991 (95% CI: 0.980–1.000) (Fig. 4F). The LOOCV accuracy was 0.940 (0.851–0.974) and Cohen's Kappa was 0.798 across the different iterations of the LOOCV procedure.

## Discussion

In the present work, we were able to independently validate the diagnostic utility of individual‐adjusted brain morphometry for the differential diagnosis of atypical parkinsonism. One of the particular strengths of the current work is the blinded evaluation of the observer‐dependent atrophy ratings and the imaging‐based classification. Notably, the differentiation of PSP from PD and MSA shows remarkably high accuracy using both a visual interpretation of automatically generated atrophy maps as well as an automated ROI classification algorithm.

Recent advancements in MRI techniques have introduced several measures to detect structural changes in the brain. Voxel‐based morphometry (VBM) is one of the most widely used neuroimaging methods that quantitatively assess differences in anatomical brain structures.^21^ It primarily focuses on group‐level comparisons, but an individual‐level analysis method has been reported for detecting hippocampal atrophy in Alzheimer's Disease. The “Voxel‐based Specific Regional Analysis system for Alzheimer's Disease” (VSRAD) has been mainly developed and used as clinical support software in Japan.^22^ This software generates statistical results of regional brain volumes, such as the hippocampus, by referencing the mean and standard deviation of a control dataset. In contrast, the iVAC approach considers the effects of age, sex, intracranial volumes, and other factors in the regression model for the reference control dataset.^15^ The previous study applied the iVAC to MSA patients, demonstrating its increased sensitivity in detecting atrophic changes compared to conventional T2‐weighted image readings by experienced neurologists. Using this approach, we examined the visual evaluation of the individual z‐score maps for both gray matter and white matter to differentiate MSA, PSP, and PD. Most PSP patients consistently showed the midbrain atrophic appearance yielding a high specificity with high agreement between two blind raters, even though one rater did not have prior training for iVAC analyses. Two patients in PSP did not display typical appearance due to smaller midbrain atrophic regions having the voxels with a *z*‐score above 2. MSA patients exhibited characteristic appearance for putamen atrophy and for the pons/MCP atrophy. These appearances were consistent with the findings of a previous report.^15^ However, our results indicated a slightly lower accuracy for MSA compared to the PSP and to the previous reports. In the previous paper utilizing iVAC, most patients exhibited pons and MCP atrophy changes (96.2%), even in MSA‐P patients.^15^ This observation was suspected to be due to the predominance of the cerebellar type in East Asia^23^ or the possibility of different susceptibility in the pons and cerebellum among distinct populations. These factors might contribute to the varying diagnostic accuracy between raters in the present study due to less specificity in putaminal atrophic changes in MSA. In addition, the number of healthy controls were larger in the previous report (n = 189) than the current study (n = 48), which may have an impact on the accuracy.

Furthermore, we examined the diagnostic potential of ROI‐based *z*‐score assessments, which facilitates an automatic decision‐making process. A number of different modalities, ROI‐based, and machine learning methods has been introduced to enhance diagnostic accuracy. Regarding the analysis using T1‐WI images, the machine learning method using a 3‐node C4.5 decision tree was applied and showed the high differentiate accuracy of 97.4% for PSP and MSA from PD,^24^ and 96.8% of MSA patients from PD.^25^ The automatic analysis pipeline of magnetic resonance parkinsonism index (MRPI) or MRPI 2.0 has been introduced in differentiating PSP.^26^, ^27^ Recent reports of MRPI 2.0 using two different cohorts showed the differentiation between PSP‐P and PD with AUC = 0.93 for training data and 0.92 for test data.^28^ In addition, AUCs in discriminating between PSP and non‐PSP parkinsonism at a clinically unclassifiable stage were 0.91 for both the pons‐to‐midbrain ratio (P/M) and the MRPI and 0.98 for the P/M 2.0 and the MRPI 2.0.^29^ Diffusion MRI has been reported to detect abnormalities of the MCP and putamen in MSA. A diffusion MRI in the MCP and putamen, can effectively discriminate between MSA and PD.^30^, ^31^ Another multicenter study involving 1002 patients, a support vector algorithm with a linear kernel was applied to differentiate between PD and atypical parkinsonism using diffusion‐weighted images and motor scores, reported an AUC of 0.962 for PD versus atypical parkinsonism and 0.897 for MSA versus PSP.^32^ In our study, we have created a new, simple index to enhance the diagnostic accuracy. This index was derived by comparing the highest *z*‐score values, indicating the greatest atrophy region, from the midbrain, representing relevant region of PSP, and either the putamen or MCP, representing relevant regions of both MSA‐P and MSA‐C. The concept behind this index is to assign the highest *z*‐scores to either the positive or negative side based on the most affected brain region related to PSP and MSA. If the midbrain has the highest *z*‐score, indicating it is the most affected brain region, the index will be positive. Conversely, if the putamen or MCP has the highest *z*‐score, the index will be placed in the negative. Notably, all indices in PSP were on the positive side, while those of all except two MSA patients were on the negative side. This dichotomy contributes to the high differentiation diagnostic potential of the algorithm with an AUC of 0.991 and the LOOCV accuracy 0.940 with kappa 0.798 for PSP. While the accuracy for MSA still requires improvement, the use of both ROI‐based automatic and visual‐based *z*‐score map assessments for PSP may be potentially useful in the clinical settings, along with a physical examination.

### Limitations

Long‐term clinical follow‐up rather than pathological confirmation was considered the gold standard diagnosis in the present paper. Consequently, the accuracy of the clinical diagnosis has an impact on the diagnostic outcome, and clinical misdiagnosis can occur even when the latest diagnostic criteria are applied.^33^ This study did not differentiate between subtypes of MSA or PSP, nor did it include other variants of PSP. The potential differences between disease subtypes when using our imaging approach needs to be investigated in further studies. While the system has the potential to be clinically useful, our results suggest that the effective use may require learning or establishing consensus on subjective decisions, and the prior probability is also crucial for using this system to increase the accuracy. Further, the accuracy may also be influenced by factors such as the performance of the MRI, including the manufacturer, magnetic field strength, noise, slice thickness, anomaly, or other abnormality in the brain, and the reliability of the segmentation pipeline or software. As these techniques continue to develop, the accuracy could further improve. Finally, although we have validated the accuracy of automated ROI analysis using LOOCV, our approach requires further validation using other cohorts with larger sample size, and comparisons with other advanced techniques, to assess its clinical utility as an easy‐to‐process approach.

## Conclusion

The present study confirms the high diagnostic accuracy of individual‐supported brain morphometry for differentiating PSP, though improvements are still needed for MSA. One of the particular strengths of the present work is the blinded assignment of an imaging‐based diagnosis mitigating various forms of bias which are common in research on diagnostic tests. The MSA results consistently characteristic of atrophic patterns that can be easily identified visually, supporting the results of a previous report from Japan. Furthermore, PSP patients consistently displayed a distinct appearance in the midbrain with high diagnostic potentials. Finally, we were able to develop a simple index to differentiate parkinsonism, which resulted in notably high diagnostic accuracy for PSP patients.

## Author Roles

(1) Research Project: A. Design, B. Organization, C. Execution; (2) Statistical Analysis: A. Design, B. Execution, C. Review and Critique; (3) Manuscript Preparation: A. Writing of the First Draft, B. Review and Critique.

K.K.: 1A, 1C, 2A, 2B, 3A

F.K.: W.P.: 1A, 1B, 1C, 2C, 3B

B.H.: 1C, 2B, 3B

A.H., C.M.: 1C, 3B

C.S., E.R.G., K.S.: 1B, 1C, 2C, 3B.

## Disclosures

**Ethical Compliance Statement:** The study was approved by the Ethics Committee of the Medical University of Innsbruck. Participants’ written informed consent was obtained prior to study inclusion. We confirm that we have read the Journal's position on issues involved in ethical publication and affirm that this work is consistent with those guidelines.

**Funding sources and Conflicts of Interest:** This work was supported by the Japan Society for the Promotion of Science (JSPS) Overseas Research Fellowship to K.K. This was also funded by Oesterreichische Nationalbank (Austrian Central Bank, Anniversary Fund, project number: 14171) and the Austrian Science Fund (FWF: Der Wissenschaftsfonds, project number: KLI82‐B00).

**Financial Disclosures for the Previous 12 Months:** The authors have nothing to report in relation to this study.

## Supporting information


**TABLE S1.** Demographics
