# Supplementary material for: A Blinded Evaluation of Brain Morphometry for Differential Diagnosis of Atypical Parkinsonism
Source: Mov Disord Clin Pract. 2024 Feb 5;11(4):381–90. doi: 10.1002/mdc3.13987 (PMC10982602; doi:10.1002/mdc3.13987)
Supplement: Supplementary file 1 — TABLE S1. Demographics [file MDC3-11-381-s001.docx]

**Supplementary Table S1. Demographics**

|  | PD | MSA | PSP | P values |
| --- | --- | --- | --- | --- |
| Count, n | 96 | 18 | 20 |  |
| Age, y | 66.3 ± 9.4 | 63.5 ± 8.0 | 67.2 ± 5.3 | 0.334^†^ |
| Sex, f/m | 31/65 | 7/11 | 7/13 | 0.853^††^ |
| Duration, y | 6.1 ± 4.4 | 2.1 ± 1.4* | 2.1 ± 1.6* | < 0.001^†^ |
| Hoehn & Yahr | 2.3 ± 0.6 | 3.4 ± 0.7* | 3 ± 0.8* | < 0.001^†^ |

Continuous variables are presented as mean ± standard deviation, while categorical variables are expressed as a number. ^†^Kruskal-Wallis test; ^††^Chi-square test; * Mann Whitney U test p < 0.05 compared with PD, with the p-values corrected for multiple comparisons using Bonferroni correction.
